# Gender differences in parieto‐frontal brain functional connectivity correlates of creativity

**DOI:** 10.1002/brb3.1196

**Published:** 2019-01-27

**Authors:** Richard Silberstein, David A. Camfield, Geoffrey Nield, Con Stough

**Affiliations:** ^1^ Centre for Human Psychopharmacology Swinburne University Melbourne Vic. Australia; ^2^ Neuro‐Insight Pty Ltd Melbourne Vic. Australia

**Keywords:** brain functional connectivity, creativity, gender differences, steady‐state visually evoked potential

## Abstract

**Introduction:**

Creativity is a complex construct that lies at the core of what has made human civilizations possible. One frequently used measure of creativity is the *Abbreviated Torrance Test for Adults* that yields an overall creativity score. In this study, we examine the relationship between the task‐related differences in brain functional connectivity and the creativity score in a male and female group of participants.

**Methods:**

Brain functional connectivity was estimated from the steady‐state visual evoked potential (SSVEP) event‐related partial coherence in a group of 27 females and 27 males while they performed a low‐demand visual vigilance task and the A‐X version of the Continuous Performance Task. Task‐related differences in brain functional connectivity (ΔFC) were correlated with the creativity score separately in the female and male groups.

**Results:**

We found that the creativity score was correlated with a parieto‐frontal ΔFC component for both the female and male groups. However, significant gender differences were observed in both the timing and the laterality of the parietal component. Females exhibited a left parietal to bilateral frontal ΔFC component correlated with creativity score and this peaked on the appearance of a target in both tasks. By contrast, males demonstrated a right parietal to bilateral frontal ΔFC component correlated with creativity score which peaked on the appearance of the letter following the targets.

**Conclusion:**

These findings are discussed in the context of the role of the Default Mode Network in creativity, and the role of gender‐related differences in cortical networks that mediate creativity.

## 1 INTRODUCTION

Creativity is a complex construct that lies at the core of what has made human civilizations possible. Notwithstanding the increasing importance of creativity at a national level (Florida, 2002, 2003), neuroscience research into creativity has only seen a significant increase in the last 10 years. Much of this research has centered on either the neural correlates of the creative process (Kounios & Beeman, 2009, 2014) or the brain functional or structural correlates of measures of creativity. This paper addresses the second aspect of creativity research concerning the question, *what are the neural correlates of creativity*? Creative thought or behavior is most commonly defined as that which is both novel–original and useful–adaptive (Feist, 1998). Two common approaches to the measurement of trait creativity have involved either a variant of the divergent thinking task (DTT) or the Torrance Tests of Creative Thinking (TTCT; Runco & Acar, 2012; Torrance, 1988). The DTT tasks typically involve producing as many ideas or solutions to a problem which has more than one solution while the TTCT involve a series of verbal and figural tests of creative ability which are assessed according to four norm‐referenced measures termed “fluency,” “flexibility,” “originality,” and “elaboration” (Goff & Torrance, 2002). The TTCT is currently the most widely used and most researched test of trait creativity and has been found to be predictive of subsequent creative achievement in longitudinal studies (Goff & Torrance, 2002).

Campbell (1960) introduced a widely quoted model of the creative process known as the “blind variation selective retention” (BVSR) model of creativity. In the BVSR model, it is assumed that the process of creativity involves the generation of a number of original thoughts that are based on novel variations of the relationships between pre‐existing thoughts (blind variation) and a process that evaluates and selectively retains only the original thought deemed most satisfactory. More recently, a number of researchers have suggested that a specific cortical network, known as the Default Mode Network (DMN) may play a principle role in the generation of new ideas, irrespective of their suitability while a “cognitive control” network is responsible for the process of selective retention (Jung, Mead, Carrasco, & Flores, 2013). The DMN, first reported by Raichle et al. (2001) is a network comprising a number of regions including the ventrolateral and ventromedial prefrontal cortex, the posterior cingulate cortex, the cuneus and the inferior parietal lobe (Buckner, Andrews‐Hanna, & Schacter, 2008). The DMN is most active when awake subjects are resting and not engaged in a cognitive task and this activity manifests as “task independent thoughts” or daydreaming (Buckner et al., 2008; Raichle et al., 2001).

The suggestion that the DMN plays a central role in the generation of new ideas has been supported by several recent studies (Beaty, Benedek, Kaufman, & Silvia, 2015; Beaty, Benedek, Silvia, & Schacter, 2016; Beaty et al., 2014; Jung et al., 2013). This role was further clarified in one of the largest and most thorough studies of the brain functional connectivity correlates of individual creativity (Beaty et al., 2018). In this study, Beaty et al. examined fMRI measures of brain functional connectivity while participants performed a divergent thinking task. The study reported a robust correlation between measures of individual creative ability and FC between core nodes of the DMN, the salience and executive networks. Interestingly, this study suggests that the creativity score was associated with increased functional connectivity between the DMN and also the fronto‐parietal executive network as well as the salience network involving the insula. While the DMN is frequently considered to be most active in the no‐task condition, the authors found that the creativity score was correlated with enhanced connectivity between the DMN and task‐positive networks such as the parieto‐frontal executive network (Beaty et al., 2018).

The role of the DMN in ideas generation suggests that disorders or conditions characterized by elevated DMN activity should be associated with creativity. One such condition is low arousal and low‐arousal states are well recognized as being conducive to creativity (Martindale, 1999). Another condition associated with elevated DMN activity and creativity is attention deficit hyperactivity disorder (ADHD; Abraham, Windmann, Siefen, Daum, & Güntürkün, 2006; White & Shah, 2006, 2011). ADHD is characterized by symptoms of inattention, impulsivity and/or hyperactivity, and is one of the most commonly diagnosed pediatric neuropsychiatric disorders affecting an estimated 3%–6% of children (Brown & Cooke, 1994). Recent research has placed the DMN at the core of ADHD symptomatology.

Two of our recent papers examined brain functional connectivity (FC) changes in an ADHD and typically developing control group of boys while they performed a high‐ and a low‐demand visual vigilance task (Silberstein, Pipingas, Farrow, Levy, & Stough, 2016; Silberstein, Pipingas, Farrow, Levy, Stough, et al., 2016). In this study, we observed a parieto‐frontal FC component with DMN‐like properties. Specifically, in the control group, the FC component was high in the low‐demand task and dropped during performance of a high‐demand task, specifically the A‐X version of the Continuous Performance Task (CPT A‐X) task. Furthermore, this parieto‐frontal FC component was correlated with the reaction time in that higher FC was associated with slower reaction times. Finally, the parieto‐frontal FC component was larger in the ADHD group. While the parieto‐frontal FC component shares these features with the behavior of the DMN, the limited spatial resolution of our scalp recording does not allow us to unequivocally identify the parietal‐frontal FC as a component of the DMN. We thus adopt a more conservative approach and refer to the parieto‐frontal FC component as a *DMN‐like* network.

It was the *DMN‐like* behavior of this parietal‐frontal network led us to consider the current study which examines the relationship between the individual creativity score and the task‐related FC changes described above. If we assume that the high‐demand to low‐demand task increase in *DMN‐like* FC reflects individual DMN activity, then given the evidence linking creativity and DMN activity, we hypothesize that higher creativity scores will be associated with larger *DMN‐like* FC increases.

We propose to test the abovementioned hypothesis separately in both a male and female group of participants. While the majority fMRI‐based neuroimaging studies of creativity have reported results based on mixed gender groups (Beaty et al., 2015, 2016, 2018, 2014; Gonen‐Yaacovi et al., 2013; Jung et al., 2013; Kounios et al., 2008) and thus not designed to address possible gender effects, some structural and functional neuroimaging studies have reported significant gender differences in the neuroanatomical and activation correlates of creativity (Abraham, 2016; Abraham, Thybusch, Pieritz, & Hermann, 2014; Ryman et al., 2014; Takeuchi et al., 2017a, 2017b). Such gender differences have also been reported in terms of structural connectivity (Ingalhalikar et al., 2014) as well as a range of cognitive tasks (AlRyalat, 2017; Bell, Willson, Wilman, Dave, & Silverstone, 2006; Cahill, 2006; Hill, Laird, & Robinson, 2014; Zaidi, 2010). If the gender differences observed in the structural and functional neuroimaging studies are also apparent in our functional connectivity data, it is possible that gender‐specific effects may be diluted in a mixed gender group. We have thus taken the conservative approach of separately considering male and female group data.

## 2 METHODS

### 2.1 Participants

Fifty‐four participants were enrolled in the study, consisting of 27 females and 27 males. Mean age and IQ details are listed in Table 1. There were no statistically significant differences in mean age (2 sample unpaired *t* test *p* = 0.23) or mean IQ (2 sample unpaired *t* test *p* = 0.69). All participants were between 18 and 41 years old and were screened for the presence of pre‐existing medical, neurological or psychiatric conditions, including epilepsy. Participants were recruited via advertisements placed around Swinburne University, Hawthorn, Victoria, Australia as well as through the research participant database associated with the Brain Sciences Institute. All testing was conducted at the Brain Sciences Institute, Swinburne University. The study was approved by the Human Research Ethics Committee of Swinburne University.

**Table 1:** Means and standard deviations for age, IQ, and Creativity Score

	Male *N* = 27	Female *N* = 27	Range
Age	27.0 (6.8)	28.9 (5.0)	18–41
WASI IQ	113.1 (10.3)	112.2 (4.7)	94–134
Creativity Score	71.7 (6.4)	73.9 (6.8)	56–90

### 2.2 Materials

The Abbreviated Torrance Test for Adults (ATTA; Goff & Torrance, 2002) is an abbreviated version of the Torrance Tests of Creative Thinking. It is a paper‐and‐pencil assessment of creative ability comprising one verbal and two figural tasks. Responses to the three tasks yield four subscores for abilities termed fluency, originality, elaboration, and flexibility, and a Creativity Score (CS) is in turn derived from the subscores. In the current study, we restrict our consideration to the brain functional connectivity correlates of the CS. In the current study, the ATTA was scored by one of the authors (DAC) as well as two additional postgraduate level research assistants who had received training in ATTA administration and scoring. Full‐scale IQ was assessed using the Wechsler Abbreviated Scale of Intelligence (WASI; Wechsler, 1999).

### 2.3 Cognitive tasks

All participants performed a low‐demand Reference task followed by the CPT A‐X task. This sequence was repeated so that the Reference task and the CPT A‐X task were each performed twice. In the reference task, participants viewed a repeated presentation of the letters A, B, C, D, and E and were required to press a microswitch on the appearance of the E. In the CPT A‐X task, participants were required to respond on the unpredictable appearance of an X that had been preceded by an A. In both tasks, the letters remained on the screen for 300 ms and were followed by a blank screen for 1.5 s. All the letters were white and presented on a black screen. The ratio of targets to nontargets was 1:4. Both the Reference task and the CPT A‐X task were 180 s in duration. Reaction time was recorded to an accuracy of 1 ms. For all tasks, a correct response to a target was defined as one that occurred no <100 ms and no more than 1.5 s after the appearance of the target (E or an X preceded by an A). Any responses outside the “correct” time intervals were defined as errors of commission, or false alarms, while failure to respond in the correct interval was defined as an error of omission.

Both tasks were presented on a computer monitor. Each letter subtended a horizontal and vertical angle of approximately 1.0° when viewed by subjects from a fixed distance of 1.3 m. The stimulus used to evoke the steady‐state visually evoked potential (SSVEP) was a spatially diffuse 13‐Hz sinusoidal flicker subtending a horizontal angle of 160° and a vertical angle of 90°, which was superimposed on the visual fields. This flicker was present throughout the task and special goggles enabled subjects to simultaneously view the cognitive task and the sinusoidal flicker. The modulation depth of the stimulus, when viewed against the background, was 45%.

### 2.4 The steady‐state visually evoked potential

One of the core EEG signal processing steps is the measurement of the evoked potential that is elicited by the continuous 13 Hz visual flicker, termed the steady‐state visually evoked potential (SSVEP). The SSVEP is determined using a methodology known as complex demodulation (Silberstein, 1995) and can be considered equivalent to applying a narrow frequency band filter to the EEG that is precisely centered at the stimulus frequency. An important advantage of the SSVEP is the relatively high signal‐to‐noise ratio. This is a consequence of the fact that many sources of artifact or interfering signals either occur over a relatively wide frequency band (e.g., muscle activity or EMG) or at low frequencies (e.g., eye movements, EOG and blinks) or high frequencies (mains interference). In all cases, the interfering signals contribute minimally to the frequency band of the SSVEP and this leads to the high signal‐to‐noise ratio. The high SSVEP signal‐to‐noise ratio has also been confirmed experimentally where known amounts of interfering signals were added to artifact‐free EEG containing the SSVEP (Gray, Kemp, Silberstein, & Nathan, 2003; Silberstein, 1995).

In this study, brain electrical activity was recorded from 64 scalp sites that included all international 10–20 positions, with additional sites located midway between 10–20 locations. The specific locations of the recording sites have been previously described (Silberstein, 2006). The average potential of both earlobes served as a reference and a nose electrode served as a ground. Brain electrical activity was amplified and bandpass filtered (3 dB down at 0.1 and 30 Hz) before digitization to 16‐bit accuracy at a rate of 400 Hz. The major features of the signal processing have been described (Silberstein, Danieli, & Nunez, 2003; Silberstein, Pipingas, Farrow, Levy, Stough, et al., 2016). Briefly, the SSVEP was determined from the smoothed 13‐Hz Fourier coefficients evaluated over 10 stimulus cycles that were cosine weighted. At the stimulus frequency of 13 Hz, thus yielding a temporal resolution of 380 ms or half the 10 cycle window width because of the cosine weighting. The cosine smoothing window was then shifted 1 stimulus cycle, and the coefficients were recalculated for this overlapping period. This process was continued until the entire 180 s of activity for each task was analyzed. An identical procedure was applied to data recorded from all 64 recording sites.

### 2.5 Measurement of functional connectivity

The functional connectivity (FC) between electrode pairs was determined using a variant of the SSVEP coherence that is termed SSVEP Event‐Related Coherence (SSVEP‐ERPC) and is based on a modification of an approach first described by Andrew and Pfurtscheller (1996) (Silberstein et al., 2003; Silberstein, Pipingas, Farrow, Levy, Stough, et al., 2016). The SSVEP‐ERPC varies between 0 and 1 and like coherence, is a normalized quantity that is not determined by the SSVEP amplitude at either electrode site. Electrode pairs with high partial coherence indicate relatively stable SSVEP phase differences between electrode pairs across trials. This occurs even though SSVEP phase differences between each of the electrodes and the stimulus may be variable across trials and is equivalent to the removal of the common contribution from the SSVEP stimulus. This means that high SSVEP partial coherence between electrodes reflects a consistent synchronization between electrodes at the stimulus frequency and is not simply a consequence of two unrelated regions increasing their response to the common visual flicker. Such synchronization reflected in the SSVEP‐ERPC is thought to reflect functional connectivity between the relevant regions and as mentioned earlier, we will use the terms “SSVEP‐ERPC” and “functional connectivity” (FC) interchangeably.

For each subject, the SSVEP Event‐Related Partial Coherence (SSVEP‐ERPC) was calculated for all 2016 distinct pairs of electrodes averaged across all correct responses in the Reference and CPT A‐X tasks. The SSVEP‐ERPC is a measure of the partial coherence between electrode pairs at the stimulus frequency eliciting the SSVEP and is based.

Functional connectivity was determined during specific 5.0‐s epochs during the Reference task FC_ref_ (*t*) and during the CPT A‐X task FC_ax_ (*t*). The 5.0‐s epoch over which FC_ref_ (*t*) was evaluated comprised an initial 300 ms period where the letter “D” was displayed followed by a 1.5 s blank screen that was in turn followed by the 300 ms appearance of the “E” followed by another 1.5‐s blank screen. The corresponding 5.0 s interval over which functional connectivity during the CPT A‐X task, FC_ax_ (*t*) was evaluated comprised the 300 ms period that the “A” was on the screen and followed 1.5 s later by the appearance of the “X.” In both cases, participants were required to respond to the appearance of the second letter in the task. Specifically, “E” in the Reference task and “X” for the CPT A‐X task. For each subject, the SSVEP‐ERPC was evaluated in both tasks across all correct trials.

The time dependent, task‐related difference in FC or ΔFC(*t*) is defined by the following equation:

$$\Delta \text{FC}\left(t\right)={\text{FC}}_{\text{ref}}\left(t\right)-{\text{FC}}_{\text{ax}}\left(t\right).$$

### 2.6 Statistical considerations

To examine the relationship between the AATA Creativity scores (CS) and ΔFC(*t*) the linear correlation between individual CS and ΔFC(*t*) were calculated for each point in time for the male and female groups. Each of these yielded 2016 time series illustrating the correlation between ΔFC(*t*) and Creativity and FC over the 5‐s epoch. To explore temporal variation in the strength of the correlation between the CS and ΔFC(*t*), we determine the number of electrode pairs where the correlation between ΔFC(*t*) and CS exceeds a predetermined value at each point in time (see Silberstein et al., 2003; Silberstein, 2006; Silberstein, Pipingas, Farrow, Levy, & Stough, 2016; Silberstein, Pipingas, Farrow, Levy, Stough, et al., 2016; Silberstein, Levy, Pipingas, & Farrow, 2017).

In the current study, we determine the number of electrode pairs where the magnitude of the correlation coefficient *r* exceeds 0.48, (|*r*|≥0.48) a threshold value corresponding to *p* = 0.01 at each point in time. Figures 2 and 3 are termed “correlation frequency curves” and comprise plots illustrating the temporal variation in the number of FC measures correlated with CS where the |*r*| threshold values are either met or exceeded.

We use a permutation test to determine the level of statistical significance associated with a given number of electrode pairs where the threshold value of |*r*| is equaled or exceeded. Although the permutation test has been described previously (Silberstein, 2006; Silberstein, Pipingas, Farrow, Levy, & Stough, 2016; Silberstein, Pipingas, Farrow, Levy, Stough, et al., 2016), we take the opportunity to describe it here for the convenience of readers. For any given point in time in the male or female group correlation frequency curves, the number ΔFC(*t*)—CS correlations equal to or exceeding the |*r*| threshold is determined and designated as *N_r_*_0_. The individual creativity scores for all participants in either the male or female groups are then randomized so that any given ΔFC(*t*) and CS are unlikely to be associated with the same individual. The number of ΔFC(*t*)—CS correlations satisfying the threshold condition is then calculated (*N_ri_*) and the process is repeated 10,000 times (*i* = 1–10,000). This enabled us to determine the probability of observing the *N_r_*_0_ correlations satisfying the threshold condition on the assumption that the Null hypothesis applies.

Finally, to account for the multiple tests conducted over the 5‐s epoch of the *Correlation frequency curve*, we applied a Bonferroni correction based on the effective number of degrees of freedom of the *Correlation frequency curve*. While there are 65 data points (13 Hz × 5 s) in both curves, these points are not all independent and thus the Bonferroni correction needs to be based on the number of degrees of freedom of the frequency curves. These were determined from the e‐folding time of the autocovariance of the correlation frequency curves (Leith, 1973). In both the male and female case the correlation frequency curves demonstrated an e‐folding time of 5 data points which is also consistent with the effective smoothing window used to determine the Fourier coefficients described in the methods section *The Steady‐State Visually Evoked Potential*.

We used the following equation (Leith, 1973) to determine the effective number of degrees of freedom of the frequency curves.

$$df=\left(\text{no}\;\text{of}\;\text{cycles}\right)/\left(2\;\times \;\text{e - folding}\;\text{time}\right)$$

In a 5 s epoch, the number of cycles is 65 and the measured e‐folding time is five cycles.

This yields an effective degrees of freedom *df* = 6.5; and thus, our criterion of *p* ≤ 0.05 must be adjusted to *p *≤ (0.05/6.5) or an adjusted criterion of *p* ≤ 7.7 × 10^−3^.

## 3 RESULTS

### 3.1 ATTA results

Means and standard deviations of scaled scores for IQ and CS are displayed in Table 1. Both males, females, and the sample as a whole were found to have creative abilities within the average range, as indicated by CS scores within the range of 56–90 (Goff & Torrance, 2002).

### 3.2 Brain Functional Connectivity Correlates of Creativity Score

**Figure 1:**
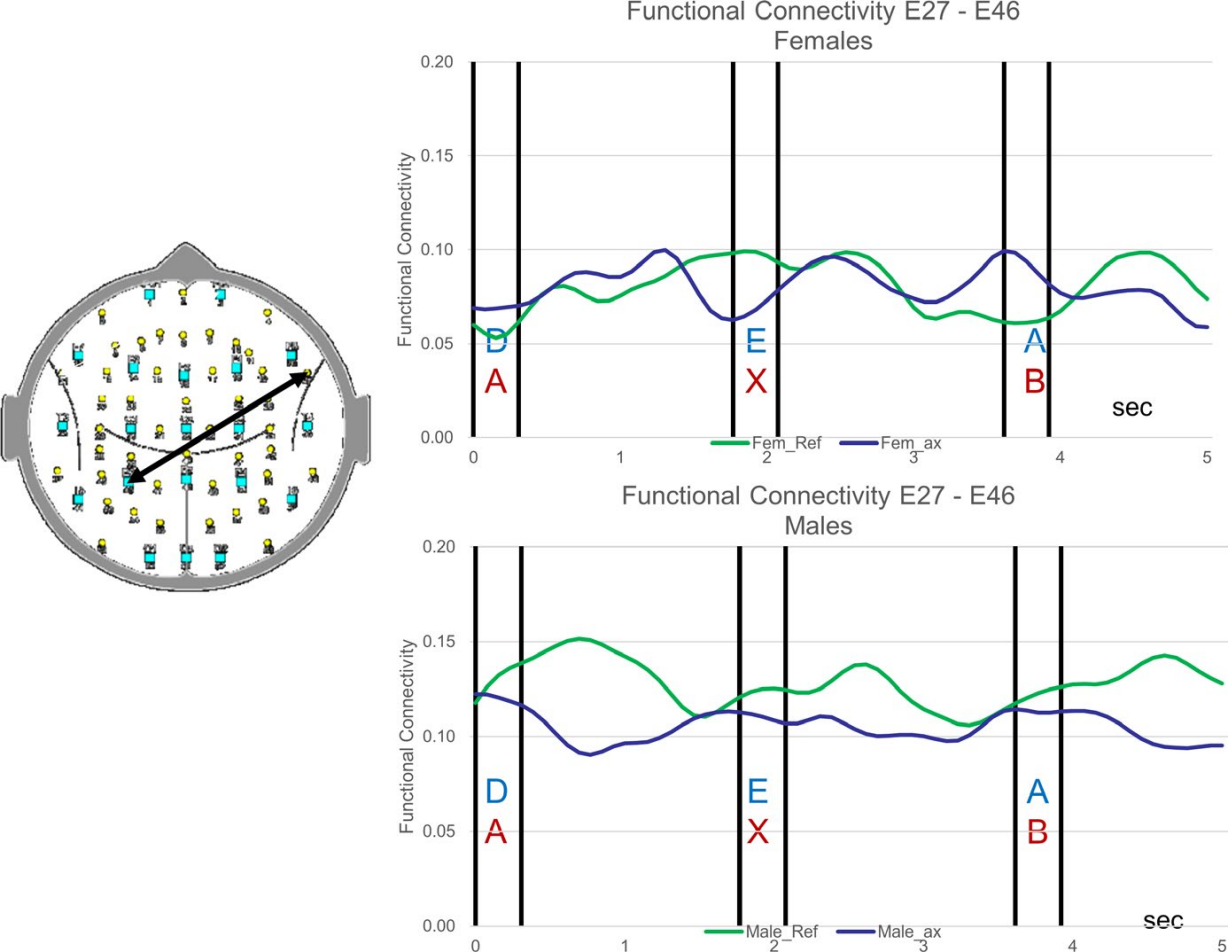
Functional connectivity between a left parietal site and right frontal site during the Reference Task (green trace) and during the CPT A‐X task (blue trace) for the female group (upper traces) and male group (lower traces). The 5‐s epochs start on the letter preceding the target letter in both tasks. For the Reference task, the trace starts on the appearance of the letter “D” while in the case of the CPT A‐X task, the trace starts on the appearance of the letter “A” that is followed by an “X”

The appearance of each letter in the Reference Task (green trace) and the CPT A‐X task (blue trace) are associated with changes in FC. The FC changes for one such electrode pair for the female and male groups are illustrated in Figure 1. The details of the task‐related changes in FC are beyond the scope of this paper and will be discussed in a subsequent publication.

In both the male and female groups, the CS was positively correlated with a parieto‐frontal ΔFC. For the female group, 110 electrode pairs exhibited a correlation between ΔFC at 1.8 s and CS that exceeded the threshold value of *r* = 0.48 and the permutation test indicated that this number of electrode pairs exceeding the threshold *r* value is significant at the level *p* = 5 × 10^−3^. In the male group, 159 electrode pairs exhibited a correlation between ΔFC at 3.8 s and CS that exceeded the threshold value of *r* = 0.48 and the permutation test indicated that this number of electrode pairs exceeding the threshold *r* value is significant at the level *p* = 3.3 × 10^−3^. Furthermore, in both the female and male cases, these finding remain statistically significant in that they satisfy the adjusted *p* = 0.05 statistical criterion of *p* ≤ 7.7 × 10^‐3^. In other words, the higher the FC during the reference task compared to the A‐X task, the higher the TTCT Creativity Score, although the point in time where when this was most prominent varied with gender. For the female group, ΔFC was most prominently correlated with CS approximately 1.8 s from the start of the epoch. For the reference task, this point in time immediately preceded the appearance of the target “E” while for the CPT A‐X task, this point in time immediately preceded the target “X” that in turn followed the earlier letter “A.” In other words, for the female group, this effect was strongest immediately before the appearance of the target in both tasks. In the male group, this effect was strongest on the appearance of a letter immediately after the appearance of the target letters in both tasks.

While both male and female groups exhibited parieto‐frontal ΔFC correlated with CS, there was a hemispheric asymmetry in this parieto‐frontal component. For the female group, we observed a left parieto‐occipital to frontal ΔFC component, by contrast, in the male group, we observed a right parieto‐temporal component to frontal ΔFC correlated with CS.

## 4 DISCUSSION

### 4.1 Creativity Score is correlated with brain functional connectivity

To the best of our knowledge, this is the first demonstration of a correlation between an SSVEP‐ERPC measure of brain functional connectivity and the ATTA CS. To the extent that the *DMN‐like* FC task‐related changes reflect individual levels of DMN activity, our findings are consistent with the growing body of research briefly reviewed in the introduction indicating a central role for the DMN in creativity. As such, our findings are consistent with the hypothesis in that both the male and female group data exhibited a positive correlation between the CS and the parieto‐frontal FC during the reference task compared to the A‐X task (or ΔFC(*t*)).

In broad terms, these findings are consistent with both the structural and functional connectivity correlates of creativity. A diffusion tensor imaging structural study of the correlation between white matter fractional anisotropy (FA), (a measure of white matter integrity) and creativity indicated that creativity was correlated with higher parieto‐frontal fiber FA (Takeuchi et al., 2010). Functional MRI studies previously reviewed also report a positive correlation between creativity scores and FC linking the DMN and parieto‐frontal executive networks, and this has been reported for both the resting state (Beaty et al., 2014) and while undertaking creative tasks (Abraham et al., 2014; Beaty et al., 2016, 2018). This relationship between creativity and parieto‐frontal functional connectivity has also been reported in EEG studies of creativity. In one of a series of EEG studies, Jaušovec and Jaušovec (2000) reported a positive correlation between parieto‐frontal alpha EEG coherence and a creativity score while participants performed dialectic problems that required high levels of creativity. Such findings are also consistent observations of parieto‐frontal high alpha and beta EEG coherence being correlated with verbal creativity scores based on the verbal Remote Association Task (Razumnikova, 2007).

While both the male and female group data supported the hypothesis in that there was a significant correlation between CS and parieto‐frontal ΔFC, there were significant gender differences in both the timing at which this correlation peaked and the hemispheric asymmetry of the ΔFC component that was correlated with CS. In the female group, the correlation peaked immediately prior to the appearance of the target and the motor responses in both the reference and A‐X tasks. By contrast, in the male group, the correlation peaked at the appearance of the letter following the target in both tasks. The hemispheric differences in the parieto‐frontal ΔFC correlated with CS were most apparent at the parieto‐occipital sites with the female group exhibiting a left parietal component and the male group a right parietal component of this FC.

We suggest these differences in the parieto‐frontal components correlated with ΔFC may reflect gender differences in cognitive strategies associated with creative thinking tasks. In the female group, the correlation is a maximum at the point in time immediately preceding the appearance of the targets, presumably a time of higher attentional demand in the tasks. By contrast, the male group correlation peaks at the appearance of the letter that can never be a target, presumably a time of lower attentional demand. We provisionally interpret this to suggest that the female creativity score is most strongly correlated with the activity of task‐related networks. By contrast, male parieto‐frontal ΔFC correlated with CS peaks at a time of lower attentional demand, a time that may coincide with higher activity in task‐independent networks such as the DMN (Buckner et al., 2008).

In the male group, the ΔFC components correlated with CS were located at right parieto‐temporal to bilateral frontal sites while the corresponding female group ΔFC components involved left occipito‐parietal and bilateral frontal sites. Once again, these hemispheric differences in the parieto‐occipital component of the ΔFC component correlated with CS are consistent with the notion that male and female groups preferentially engage different cortical networks during creative cognition.

Our observation of a gender‐based difference in CS‐ΔFC is consistent with other studies reporting a gender‐based difference in neuroimaging studies of the resting state (Filippi et al., 2013) as well as during cognitive tasks (Bell et al., 2006; Hill et al., 2014; Speck et al., 2000). Gender differences in both the structural and function neuroimaging correlates of creativity have also been reported (Abraham, 2016; Abraham et al., 2014; Ryman et al., 2014; Takeuchi et al., 2017a, 2017b) and such gender‐based differences have also been observed in EEG studies (Fink & Neubauer, 2006; Razumnikova, 2004). Apart from suggesting that females and male TTCT creativity scores are mediated by different patterns of cortical networks, these findings also suggest that caution should be exercised in pooling male and female participants as such pooling will dilute significant gender differences.

### 4.2 Creativity and ADHD

**Figure 2:**
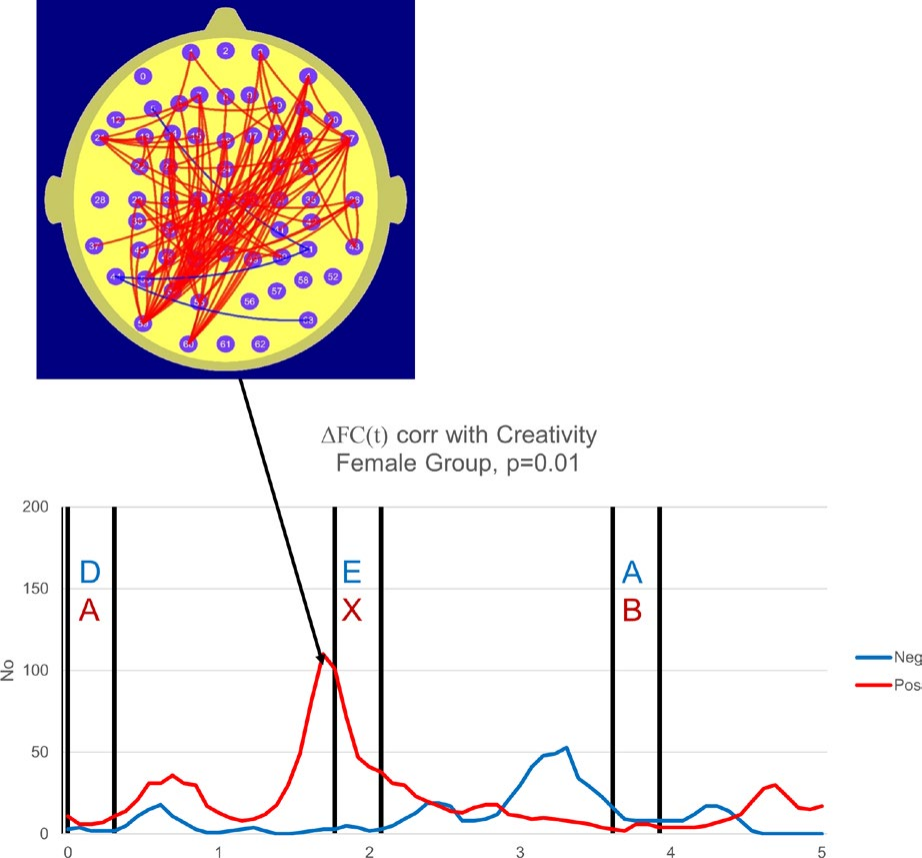
Number and location of electrode pairs where ΔFC(*t*) (functional connectivity during the low‐demand reference task minus the functional connectivity during the CPT A‐X task or ΔFC(*t*) = FC_ref_ (*t*) − FC_ax_ (*t*)) was correlated with the Creativity Score (CS) at the |*r*| > 0.48 level. The red graph illustrates the number of electrode pairs where ΔFC(*t*) is positively correlated with CS while the blue trace illustrates the number of electrode pairs where ΔFC(*t*) is negatively correlated with CS. This figure illustrates the topography of the correlated ΔFC(*t*) measures for the female group

**Figure 3:**
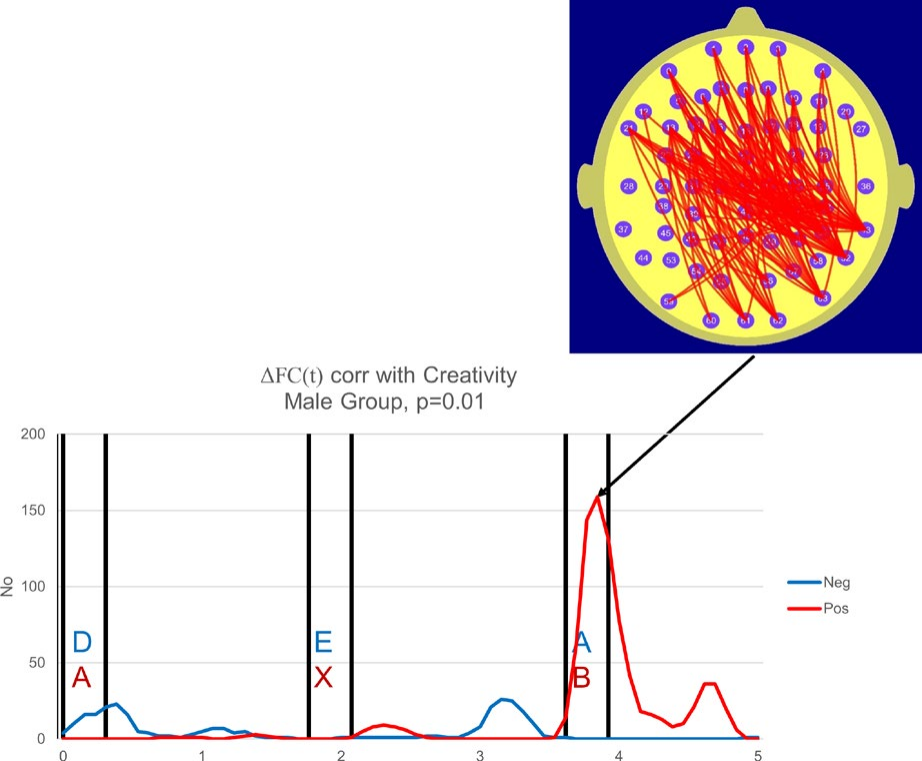
Number and location of electrode pairs where ΔFC(*t*) (functional connectivity during the low‐demand reference task minus the functional connectivity during the CPT A‐X task or ΔFC(*t*) = FC_ref_ (*t*) − FC_ax_ (*t*)) was correlated with the Creativity Score (CS) at the |*r*| > 0.48 level. The red graph illustrates the number of electrode pairs where ΔFC(*t*) is positively correlated with CS while the blue trace illustrates the number of electrode pairs where ΔFC(*t*) is negatively correlated with CS. This figure illustrates the topography of the correlated ΔFC(*t*) measures for the male group

Our data may also be of relevance to the issue of the relationship between Attention Deficit Hyperactivity Disorder (ADHD) and creativity mentioned in the introduction. It is important to appreciate that ADHD is being increasingly viewed as a network disorder with the ADHD symptomatology now considered to be a consequence of abnormal DMN activity (Castellanos, Sonuga‐Barke, Milham, & Tannock, 2006; Silberstein et al., 2017). We have previously reported increased parieto‐frontal parietal FC that appeared to reflect DMN activity in an ADHD group performing the CPT A‐X task (Silberstein, Pipingas, Farrow, Levy, & Stough, 2016; Silberstein, Pipingas, Farrow, Levy, Stough, et al., 2016). Interestingly, the parieto‐frontal FC component correlated with CS described in the current study (Figures 2 and 3) has a similar topography to the parieto‐frontal FC component, we observed in the ADHD studies referred to above (Silberstein, Pipingas, Farrow, Levy, Stough, et al., 2016 see figure 3b, 5b and 5c) and we suggest that this similarity is consistent with the notion of an association between creativity and ADHD (Abraham et al., 2006; White & Shah, 2006, 2011).

This association between creativity, considered a desirable cognitive attribute, with ADHD, a condition normally considered in terms of cognitive deficits opens a broader question of the way ADHD should be considered. While ADHD is generally considered to comprise a set of cognitive deficits, there is growing evidence that ADHD may have had an evolutionary survival advantage, especially in hunter‐gatherer societies. This was first suggested by Hartmann (1993) and more recent studies of the survival advantage in hunter‐gatherer societies of the dopamine receptor DR4 polymorphism that is implicated in ADHD appears to support this association (Eisenberg, Campbell, Gray, & Sorenson, 2008; Gizer, Ficks, & Waldman, 2009).

Given the link between creativity and ADHD as well as evidence that some of the genetic correlates of ADHD appear to confer survival advantages in hunter‐gatherer societies, it may be time to reconsider the current notion of ADHD as simply a cognitive deficit. Intellectual creativity is now considered one of the most important drivers of future economic well‐being of nations. The fact that ADHD is associated with creativity as well offering survival advantages suggests that there may be value in reconsidering ADHD as a particular “mode of thought” rather than simply a “disorder.” Thus, while this ADHD “mode of thought” has its well‐recognized associated disadvantages, it also confers significant potential advantages in terms of creativity. This opens a wider question beyond the scope of this paper has psychiatry pathologized a mode of thought, we associate with ADHD?

## 5 CONCLUDING COMMENTS AND STUDY LIMITATIONS

In concluding, it is appropriate to comment on the study limitations. The major limitation stems from the limited spatial resolution of the scalp recordings of brain electrical activity. Thus, while our FC findings are consistent with the behavior of the DMN, the low spatial resolution of the scalp recording makes it inappropriate to unambiguously identify the FC component with the DMN. As mentioned in the introduction, we have acknowledged this limitation and refer the FC component as having *DMN‐like* properties.

Notwithstanding the consistency of our findings with fMRI and EEG studies of creativity, these findings need to be confirmed in larger and ideally, independent studies. In considering such replications, it is important to consider some of our specific findings. The first one flows from our observations of significant gender differences in the CS‐FC. Our findings suggest it is not appropriate to pool male and female findings. Many studies examining the relationship between brain activity and creativity pool male and female data, possibly diluting some interesting gender‐specific effects. Finally, in considering a replication study, it is important to note that the findings depend very much on the methodology used to determine FC. Our method using the 13 Hz SSVEP to determine FC will be biased toward cortical communication components mediated by oscillations around 13 Hz. This is important as “top‐down” or “feedback” cortical communication is thought to be mediated by synchronous oscillations in the 10–20 Hz range and thus our findings are preferentially sensitive to top‐down processes (Fries, 2015; Silberstein, Pipingas, Farrow, Levy, & Stough, 2016).

## CONFLICT OF INTEREST

Authors Richard Silberstein and Geoffrey Nield were employed by and hold stock in Neuro‐Insight Pty Ltd, none of the other authors have a conflict of interest to declare.
